# Hearing impairment and cognitive function among a community-dwelling population in Japan

**DOI:** 10.1186/1744-859X-10-27

**Published:** 2011-10-01

**Authors:** Norio Sugawara, Akira Sasaki, Norio Yasui-Furukori, Seiji Kakehata, Takashi Umeda, Atsushi Namba, Shigeyuki Nakaji, Hideichi Shinkawa, Sunao Kaneko

**Affiliations:** 1 Department of Psychiatry, Hirosaki-Aiseikai Hospital, Hirosaki, 036-8151, Japan; 2 Department of Neuropsychiatry, Hirosaki University School of Medicine, Hirosaki, 036-8562, Japan; 3 Department of Otorhinolaryngology, Hirosaki University School of Medicine, Hirosaki, 036-8562, Japan; 4 Department of Social Medicine, Hirosaki University School of Medicine, Hirosaki, 036-8354, Japan

## Abstract

**Background:**

Hearing impairment is a prevalent and chronic condition in older people. This study investigated the relationship between cognitive function and hearing impairment in a Japanese population.

**Methods:**

A pure-tone average (0.5-2.0 kHz) was used to evaluate hearing impairment in 846 participants of the Iwaki Health Promotion Project who were aged at least 50 years old (310 men and 536 women). We also administered the Mini-Mental State Examination (MMSE), the Center for Epidemiologic Studies for Depression (CES-D) scale, Starkstein's apathy scale (AS) and the Short Form Health Survey Version 2 (SF-36v2). A multiple linear regression analysis assessed the association between hearing impairment and mental correlates.

**Results:**

The overall prevalence of hearing impairment in this study population was 37.7%. The participants with hearing impairment were older and less educated compared to those with no hearing problems. We observed significant differences in the MMSE and AS scores between the mild/moderate to severe groups versus the non-impaired group. After adjusting for age, gender and amount of education, hearing impairment was significantly associated with MMSE and AS scores, but not with CES-D scores. Hearing impairment was significantly related to the social functioning (SF) and role emotional (RE) scores of the SF-36v2.

**Conclusions:**

Hearing impairment is common among older people and is associated with cognitive impairment, apathy and a poor health-related quality of life. Screening for and correcting hearing impairments might improve the quality of life and functional status of older patients.

## Introduction

Age-related hearing impairment is a prevalent yet under-recognized health issue [1,2]. Previous studies [2-4] have reported a high prevalence of hearing impairment (between 35% and 45%) among older people. Hearing impairment is associated with decreased physical functioning, psychosocial impairments [5], increased social isolation [6], health condition [7] and health-related quality of life [8,9].

The association between hearing impairment and cognitive function has been recognized for many years [10]. In a case-control study, Uhlmann *et al*. [11] reported that greater hearing impairment was associated with a higher probability of dementia. Furthermore, these authors stated that loss of sensory input due to hearing impairment might cause cognitive decline.

Some studies have also suggested that there is an association between hearing impairment and depression [1,12], whereas other studies have found no such association [13,14]. Differences in the samples and methods used as well as an inability to adjust for numerous potential confounders might explain these conflicting results. The coexistence of apathy, defined as reduced motivation or lack of initiative and motivation, among patients with late-onset depression, might also explain these differences [15,16]. Apathy may be confused with depression because both conditions feature loss of interest and initiative, fatigue and poor executive function in their symptomatologies [17]. Although several reports have mentioned that hearing impairment may increase the risk of depression, data are lacking regarding the relationship between hearing impairment and apathy.

The present study sought to clarify the relationship between hearing impairment and cognitive function in an older Japanese population. We also assessed the relationship between hearing impairment and apathy. To the best of our knowledge, this study is the largest to date to evaluate the association between hearing impairment and cognitive function in Japan.

## Methods

### Participants

The study was conducted between June 2008 and June 2009. We recruited 846 volunteers who were at least 50 years old (310 men and 536 women) and had participated in the Iwaki Health Promotion Project. The Ethics Committee of the Hirosaki University School of Medicine approved the data collection for this study. All participants provided written informed consent prior to the study. We obtained demographic data (age, gender, amount of education) using self-questionnaires and interviews.

### Hearing impairment assessment

A conventional audiometer (AA-73A, RION Co., Ltd. Tokyo, Japan) obtained air-conduction pure-tone thresholds in both ears at 500, 1, 000 and 2, 000 Hz. Using an average threshold over three frequencies for the better ear, we defined mild hearing impairment as participants who could not hear below 40 dB (to 25 dB) and moderate to severe hearing impairment as those who could not hear at higher thresholds than 40 dB.

### Assessment of mental correlates

The Mini-Mental State Examination (MMSE) was used to measure the participants' global cognitive status by assessing their orientation to place and time, short-term memory and episodic long-term memory, as well as their ability in subtraction, sentence construction and oral language ability. The maximum score on the MMSE is 30 [18].

To quantify apathy, we used the Japanese version of the Apathy Scale (AS) by Starkstein *et al*. [19-21]. The AS is a 14-item self-report scale that measures spontaneity, initiation, emotionality, activity level, and interest in hobbies. Answers were scored against four grades (0-3), and the total score was used for the analysis. The most reliable results were obtained at a cut-off score of 16 points.

The Center for Epidemiologic Studies for Depression (CES-D) scale was used to measure participants' depressive status [22,23]. This questionnaire has been used widely to measure depressive symptoms and screen for depression [24,25]. The CES-D is a 20-item self-report measure that focuses on depressive symptoms over the previous week. The maximum score is 60, and lower scores are associated with greater depression.

The Short Form Health Survey Version 2 (SF-36v2) was used to assess participants' health-related quality of life (HRQOL). The SF-36v2 is a standardized international 36-item self-administered questionnaire that was translated, adapted, and validated for use in Japan [26]. This measures eight QOL domains of health status: physical functioning (PF), role physical (RP), bodily pain (BP), general health perception (GH), vitality (VT), social functioning (SF), role emotional (RE), and mental health (MH). For each QOL domain, a score ranging from 0 to 100 is calculated; higher scores indicate the higher perceptions of HRQOL. In addition, scores in all eight domains are combined to calculate more comprehensive indicators of physical and mental health: the Physical Component Summary (PCS), and the Mental Component Summary (MCS). The PCS and MCS are standardized (Japanese average = 50, standard deviation = 10) to compare to the general population or to results of other studies.

### Statistical analysis

We computed descriptive statistics to describe the demographic and clinical variables. A one-way analysis of variance (ANOVA) was used to compare demographic and clinical characteristics between groups. The Dunnet test was used for post hoc comparisons. Pearson's correlation analysis was used to assess the relationship between SF-36v2 scores and hearing impairment. After adjusting for confounding demographic factors (age, gender and amount of education), a multiple linear regression analysis was used to examine the relationships between hearing loss and MMSE, CES-D, AS and SF-36v2 scores. We considered a value of *P *< 0.05 to be significant. We analyzed the data using PASW Statistics (v. 18) for Windows(SPSS Inc., Chicago, IL, USA).

## Results

### Demographic characteristics

The average hearing thresholds for participants who were 50-59, 60-69 and ≥70 years old were 19.9 ± 7.0 dB, 23.5 ± 8.0 dB and 29.4 ± 11.6 dB, respectively. We divided the participants into three groups according to their level of hearing impairment thresholds: none (< 25 dB), n = 527; mild (25-39 dB), n = 265; and moderate to severe (> 39 dB), n = 54. The prevalence of hearing impairment in our sample was 37.7%. Table 1 lists the participants' clinical characteristics. The participants with hearing impairment were older and less educated compared to those with no hearing problems. In addition, we observed significant differences in the MMSE score between the mild/moderate to severe groups versus the non-impaired group. The mild hearing impairment group had higher AS scores than the non-impaired group, whereas the moderate to severe hearing impairment group did not.

**Table 1:** Demographic characteristics of the subjects

	Total, n = 846	Hearing impairment			ANOVA *P *value
			
		None (-24 dB), n = 527	Mild (25-39 dB), n = 265	Moderate to severe (-40 dB), n = 54	
Age	63.9 ± 8.3	61.3 ± 7.4	67.3 ± 7.9^a^	72.6 ± 6.7^a^	< 0.001
Amount of education	10.8 ± 2.2	11.3 ± 2.0	10.1 ± 2.2^a^	9.1 ± 2.2^a^	< 0.001
MMSE score	27.8 ± 2.6	28.3 ± 2.1	27.1 ± 2.8^a^	25.6 ± 3.5^a^	< 0.001
CES-D score	9.7 ± 6.1	9.5 ± 6.1	10.0 ± 6.3	10.0 ± 5.3	0.536
Apathy scale score	13.9 ± 6.3	13.4 ± 6.2	14.6 ± 6.5^a^	15.2 ± 6.4^b^	< 0.05

Data are presented as mean ± SD.

^a^Indicates a significant difference (*P *< 0.05) from no hearing impairment group.

^b^Indicates a statistical trend with no hearing impairment group (*P *< 0.10).

ANOVA = analysis of variance; CES-D = Center for Epidemiologic Studies for Depression scale; MMSE = Mini-Mental State Examination.

### SF-36v2 scores by degree of hearing impairment

Table 2 shows the SF-36v2 scores by degree of hearing impairment. We observed significant differences in PF, RP and PCS scores when we compared the mild and moderate to severe groups to the each auditory status subgroup was compared to the non-impaired group. The mild hearing impairment group showed lower BP scores and higher MCS scores compared to the non-impaired group, whereas the moderate to severe hearing impairment group did not. SF and RE scores were lower for the non-impaired group compared to the moderate to severe hearing impairment group.

**Table 2:** Short Form 36 (SF-36) scores by degree of hearing impairment

	Total, n = 846	Hearing impairment			ANOVA *P *value
			
		None (-24 dB), n = 527	Mild (25-39 dB), n = 265	Moderate to severe (-40 dB), n = 54	
PF	80.7 ± 19.7	83.4 ± 18.7	76.6 ± 19.9^a^	74.4 ± 23.2^a^	< 0.001
RP	87.2 ± 20.5	89.1 ± 19.3	85.2 ± 21.1^a^	78.7 ± 24.4^a^	< 0.001
BP	71.3 ± 22.7	72.9 ± 22.4	68.7 ± 23.1^a^	67.8 ± 21.8	< 0.05
GH	60.4 ± 17.6	60.5 ± 16.9	60.1 ± 19.1	61.2 ± 16.5	0.912
VT	65.8 ± 19.7	65.5 ± 19.4	67.1 ± 19.6	62.2 ± 22.0	0.210
SF	90.7 ± 17.2	91.2 ± 16.5	91.1 ± 16.9	83.3 ± 23.4^a^	< 0.01
RE	89.7 ± 19.2	90.9 ± 18.0	89.4 ± 19.1	79.3 ± 26.7^a^	< 0.001
MH	76.2 ± 17.7	75.8 ± 17.5	76.7 ± 18.2	76.9 ± 17.2	0.742
PCS	46.3 ± 12.4	48.0 ± 11.7	44.1 ± 12.3^a^	40.2 ± 14.8^a^	< 0.001
MCS	53.0 ± 9.0	52.4 ± 8.9	54.1 ± 9.2^a^	53.8 ± 9.7	< 0.05

Data are presented as mean ± SD.

^a^Indicates a significant difference (*P *< 0.05) from no hearing impairment group.

ANOVA = analysis of variance; BP = bodily pain; GH = general health perception; MCS = Mental Component Summary; MH = mental health; PCS = Physical Component Summary; PF = physical functioning; RE = role emotional; RP = role physical; SF = social functioning; VT = vitality.

### Multiple regression analysis for mental correlates

Table 3 details the multiple regression analysis for the MMSE, CES-D and AS scores in association with age, gender and amount of education. MMSE and AS scores were independently and significantly associated with hearing impairment.

**Table 3:** Multiple regression analysis for mental correlates

Independent variables		Beta coefficient	t Value	*P *value
MMSE	Age	-0.154	-4.220	< 0.001
	Gender	-0.170	-5.688	< 0.001
	Education	0.320	9.174	< 0.001
	Hearing level	-0.141	-4.177	< 0.001
CES-D score	Age	-0.058	-1.355	0.176
	Gender	-0.067	-1.915	0.056
	Education	-0.108	-2.652	< 0.01
	Hearing level	0.026	0.650	0.516
Apathy scale	Age	-0.087	-2.065	< 0.05
	Gender	-0.041	-1.183	0.237
	Education	-0.139	-3.476	< 0.01
	Hearing level	0.094	2.434	< 0.05

CES-D = Center for Epidemiologic Studies for Depression scale.

### The relationship between SF-36v2 scores and hearing impairment

Table 4 shows the single and multiple correlations between hearing impairment and SF-36v2 scores. We observed significant correlations between hearing level and the PF, RP, BP, SF, RE, PCS and MCS scores. After adjusting for age, gender and amount of education, the SF and RE scores were significantly related to hearing impairment.

**Table 4:** Multiple correlation between Short Form 36 (SF-36) and hearing level

	Single correlation		Multiple correlation				
	
	r	*P *value	B	SE	t Value	Beta	*P *value
PF	-0.173	< 0.001	-0.059	0.074	-0.790	-0.029	0.430
RP	-0.154	< 0.001	-0.082	0.079	-1.028	-0.038	0.304
BP	-0.091	< 0.01	-0.168	0.092	-1.828	-0.071	0.068
GH	-0.018	0.593	-0.042	0.071	-0.582	-0.023	0.561
VT	-0.006	0.852	-0.129	0.079	-1.628	-0.063	0.104
SF	-0.096	< 0.01	-0.151	0.070	-2.165	-0.084	< 0.05
RE	-0.154	< 0.001	-0.177	0.076	-2.332	-0.089	< 0.05
MH	0.011	0.751	-0.121	0.071	-1.698	-0.066	0.090
PCS	-0.208	< 0.001	-0.054	0.046	-1.186	-0.042	0.236
MCS	0.072	< 0.05	-0.061	0.035	-1.730	-0.065	0.084

Multiple model included age, gender and duration of education as confounders.

BP = bodily pain; GH = general health perception; MCS = Mental Component Summary; MH = mental health; PCS = Physical Component Summary; PF = physical functioning; RE = role emotional; RP = role physical; SF = social functioning; VT = vitality.

## Discussion

This study evaluated the association between hearing impairment and cognitive function among a community-dwelling population in Japan. The prevalence rate of hearing impairment in this sample was 37.7%; furthermore, we observed an age-related increase of hearing thresholds among participants. We found a significant association between hearing impairment and cognitive function using the MMSE. Hearing impairment was also significantly related to AS scores, but not to CES-D scores.

Previous studies have shown that hearing ability predicts cognitive function. Multiple cross-sectional studies [12,27,28] have found a significant relationship between hearing impairment and cognitive function, even after adjusting for confounding factors. Furthermore, in longitudinal study on women who were at least 69 years old, Lin *et al*. [29] demonstrated that hearing impairment had tendency to associate with cognitive decline (OR 1.38, 95% CI 0.95 to 2.00).

Associations between hearing impairment and depression have also been reported. Some cross-sectional studies [1,12,30,31] have found a positive correlation between hearing impairment and depression. However, Gopinath *et al*. [14] did not find the association between hearing impairment and depression using the CES-D scale in participants who were at least 60 year old in the Blue Mountain study. In addition, Chou [13] did not find an association between hearing impairment and depression in the English Longitudinal Study of Aging in participants who were at least 50 year old. We found a relationship between hearing impairment related with apathy, but not with then CES-D scale. Previous studies [32,33] have suggested that apathy and depression have different etiologies in older people. Depression measurements often include items that evaluate apathy. Therefore, these depression measures might overestimate depression and underestimate of apathy [34-36].

In this study, hearing impairment was significantly associated with adjusted scores of SF-36v2 standardized scores in social functioning (SF) and role emotional (RE) domains. These results suggest that people with hearing impairment might experience more negative emotional reaction and social functioning limitations compared to participants without hearing problems. Conversely, previous studies [8,9] have noted that five or six domains of the SF-36 scores are related to hearing impairment after adjusting for confounders. The differences in these findings might be due to differences in sample size or ethnic group.

The mechanisms for the association between hearing impairment and cognitive function are not clear. One possible explanation for this relationship is that reductions in the quality or quantity of auditory input lead to structural or functional changes in the brain, which results in a decline of cognitive function. In this study, we found a relationship between hearing impairment and SF and RE scores on the SF-36v2. These aspects might cause cognitive decline or apathy. Another explanation might be that brain damage causes both hearing impairment and decline of mental function in older people. We did not image the brains of our participants, therefore we cannot evaluate this possibility.

The current study has several limitations. First, this study was cross-sectional study, so we cannot determine whether hearing impairment causes cognitive decline and apathy. To do so, a follow-up survey will be necessary. Second, the MMSE was primarily administered verbally; however, one component requires participants to copy an overlapping pentagon. This section might be biased against participants with severe hearing and vision impairments. Third, because all participants were volunteers with interests in their health, they may be healthier than the general population. Thus, those not in the study may have poorer cognitive outcomes [37]. This 'selective bias' must also be considered in studies of older populations. Although people with severe hearing impairments may not live to old age [38], our findings indicate that hearing impairment is an independent risk factor of cognitive decline.

## Conclusions

Hearing impairment is common among older people, and it is associated with cognitive impairment, apathy and poorer HRQOLs. Hearing impairment screening for older patients may not only improve patients' short-term QOL, but also identify those who are at increased risk for future cognitive decline and apathy. From a preventive standpoint, there is growing evidence that correcting hearing impairments can improve QOL and functional status in older people.

## Competing interests

The authors declare that they have no competing interests.

## Authors' contributions

NS conceived the study, designed the study, conducted the statistical analysis, interpreted the data and wrote the initial draft of the manuscript. SK had full access to all of the data in the study and takes responsibility for the integrity of the data and the accuracy of the data analysis. AS, HS and NYF contributed to study design and assisted in drafting the manuscript. TU and SN completed initial survey construction, recruitment of participants. SK and AN participated in the data collection and the interpretation of the results. All authors have approved the manuscript.

## References

[B1] Naramura H Nakanishi N Tatara K Ishiyama M Shiraishi H Yamamoto A Physical and mental correlates of hearing impairment in the elderly in Japan Audiology 1999 38 24 29 10.3109/00206099909072999 10052833

[B2] Sakamoto N Sasaki A Kakehata S Futai K Namba A Shinkawa H Takahashi I Matsuzaka M Hearing investigation in the Iwaki Health Promotion Project [in Japanese] Audiology Japan 2008 51 270 278 10.4295/audiology.51.270

[B3] Cruickshanks KJ Wiley TL Tweed TS Klein BE Klein R Mares-Perlman JA Nondahl DM Prevalence of hearing loss in older adults in Beaver Dam, Wisconsin. The Epidemiology of Hearing Loss Study Am J Epidemiol 1998 148 879 886 9801018 10.1093/oxfordjournals.aje.a009713

[B4] Reuben DB Walsh K Moore AA Damesyn M Greendale GA Hearing loss in community-dwelling older persons: national prevalence data and identification using simple questions J Am Geriatr Soc 1998 46 1008 1011 9706892 10.1111/j.1532-5415.1998.tb02758.x

[B5] Bess FH Lichtenstein MJ Logan SA Burger MC Nelson E Hearing impairment as a determinant of function in the elderly J Am Geriatr Soc 1989 37 123 128 2910970 10.1111/j.1532-5415.1989.tb05870.x

[B6] Weinstein BE Ventry IM Hearing impairment and social isolation in the elderly J Speech Hear Res 1982 25 593 599 7162161 10.1044/jshr.2504.593

[B7] Crews JE Campbell VA Vision impairment and hearing loss among community-dwelling older Americans: implications for health and functioning Am J Public Health 2004 94 823 829 10.2105/AJPH.94.5.823 15117707 PMC1448344

[B8] Dalton DS Cruickshanks KJ Klein BE Klein R Wiley TL Nondahl DM The impact of hearing loss on quality of life in older adults Gerontologist 2003 43 661 668 10.1093/geront/43.5.661 14570962

[B9] Chia EM Wang JJ Rochtchina E Cumming RR Newall P Mitchell P Hearing impairment and health-related quality of life: the Blue Mountains Hearing Study Ear Hear 2007 28 187 195 10.1097/AUD.0b013e31803126b6 17496670

[B10] Herbst KG Humphrey C Hearing impairment and mental state in the elderly living at home Br Med J 1980 281 903 905 10.1136/bmj.281.6245.903 7427503 PMC1714179

[B11] Uhlmann RF Larson EB Rees TS Koepsell TD Duckert LG Relationship of hearing impairment to dementia and cognitive dysfunction in older adults JAMA 1989 261 1916 1919 10.1001/jama.261.13.1916 2926927

[B12] Cacciatore F Napoli C Abete P Marciano E Triassi M Rengo F Quality of life determinants and hearing function in an elderly population: Osservatorio Geriatrico Campano Study Group Gerontology 1999 45 323 328 10.1159/000022113 10559650

[B13] Chou KL Combined effect of vision and hearing impairment on depression in older adults: evidence from the English Longitudinal Study of Ageing J Affect Disord 2008 106 191 196 10.1016/j.jad.2007.05.028 17602753

[B14] Gopinath B Wang JJ Schneider J Burlutsky G Snowdon J McMahon CM Leeder SR Mitchell P Depressive symptoms in older adults with hearing impairments: the Blue Mountains Study J Am Geriatr Soc 2009 57 1306 1308 10.1111/j.1532-5415.2009.02317.x 19570163

[B15] Starkstein SE Fedoroff JP Price TR Leiguarda R Robinson RG Apathy following cerebrovascular lesions Stroke 1993 24 1625 1630 10.1161/01.STR.24.11.1625 8236333

[B16] Kobayashi S Epidemiology of vascular depression [in Japenese] Depress Front 2003 1 15 21

[B17] Fones CS Distinguishing apathy syndromes from vascular depression Arch Gen Psychiatry 1998 55 844 845 10.1001/archpsyc.55.9.844 9736012

[B18] Folstein MF Folstein SE McHugh PR "Mini-mental state". A practical method for grading the cognitive state of patients for the clinician J Psychiatr Res 1975 12 189 198 10.1016/0022-3956(75)90026-6 1202204

[B19] Starkstein SE Mayberg HS Preziosi TJ Andrezejewski P Leiguarda R Robinson RG Reliability, validity, and clinical correlates of apathy in Parkinson's disease J Neuropsychiatry Clin Neurosci 1992 4 134 139 1627973 10.1176/jnp.4.2.134

[B20] Okada K Kobayashi S Yamagata S Takahashi K Yamaguchi S Poststroke apathy and regional cerebral blood flow Stroke 1997 28 2437 2441 10.1161/01.STR.28.12.2437 9412628

[B21] Yamagata S Yamaguchi S Kobayashi S Impaired novelty processing in apathy after subcortical stroke Stroke 2004 35 1935 1940 10.1161/01.STR.0000135017.51144.c9 15205493

[B22] Radloff LS The CES-D scale: a self-report depression scale for research in the general population Appl Psychol Meas 1977 1 385 401 10.1177/014662167700100306

[B23] Shima S Shikano T Kitamura T New self-rating scales for depression [in Japanese] Clin Psychiatry 1985 27 717 723

[B24] Zich JM Attkisson CC Greenfield TK Screening for depression in primary care clinics: the CES-D and the BDI Int J Psychiatry Med 1990 20 259 277 10.2190/LYKR-7VHP-YJEM-MKM2 2265888

[B25] Sugawara N Yasui-Furukori N Sasaki G Umeda T Takahashi I Danjo K Matsuzaka M Kaneko S Nakaji S Assessment of the Center for Epidemiological Studies Depression Scale factor structure among middle-aged workers in Japan Psychiatry Clin Neurosci 2011 65 109 111 10.1111/j.1440-1819.2010.02172.x 21265946

[B26] Funahashi K Takahashi I Danjo K Matsuzaka M Umeda T Nakaji S Smoking habits and health-related quality of life in a rural Japanese population Qual Life Res 2011 20 199 204 10.1007/s11136-010-9748-8 20857336

[B27] Tay T Wang JJ Kifley A Lindley R Newall P Mitchell P Sensory and cognitive association in older persons: findings from an older Australian population Gerontology 2006 52 386 394 10.1159/000095129 16921251

[B28] Cutler SJ Grams AE Correlates of self-reported everyday memory problems J Gerontol 1988 43 S82 90 3361099 10.1093/geronj/43.3.s82

[B29] Lin MY Gutierrez PR Stone KL Yaffe K Ensrud KE Fink HA Sarkisian CA Coleman AL Mangione CM Study of Osteoporotic Fractures Research Group. Vision impairment and combined vision and hearing impairment predict cognitive and functional decline in older women J Am Geriatr Soc 2004 52 1996 2002 10.1111/j.1532-5415.2004.52554.x 15571533

[B30] Abrams TE Barnett MJ Hoth A Schultz S Kaboli PJ The relationship between hearing impairment and depression in older veterans J Am Geriatr Soc 2006 54 1475 1477 10.1111/j.1532-5415.2006.00875.x 16970669

[B31] Ishine M Okumiya K Matsubayashi K A close association between hearing impairment and activities of daily living, depression, and quality of life in community-dwelling older people in Japan J Am Geriatr Soc 2007 55 316 317 10.1111/j.1532-5415.2007.01067.x 17302679

[B32] van der Mast RC Vinkers DJ Stek ML Bek MC Westendorp RG Gussekloo J de Craen AJ Vascular disease and apathy in old age. The Leiden 85-Plus Study Int J Geriatr Psychiatry 2008 23 266 271 10.1002/gps.1872 17621380

[B33] Sugawara N Yasui-Furukori N Umeda T Kaneda A Sato Y Takahashi I Matsuzaka M Danjo K Nakaji S Kaneko S Ankle brachial pressure index as a marker of apathy in a community-dwelling population Int J Geriatr Psychiatry 2011 26 409 414 10.1002/gps.2541 20658477

[B34] Landes AM Sperry SD Strauss ME Prevalence of apathy, dysphoria, and depression in relation to dementia severity in Alzheimer's disease J Neuropsychiatry Clin Neurosci 2005 17 342 349 10.1176/appi.neuropsych.17.3.342 16179656

[B35] Marin RS Firinciogullari S Biedrzycki RC The sources of convergence between measures of apathy and depression J Affect Disord 1993 28 7 14 10.1016/0165-0327(93)90072-R 8326082

[B36] Levy ML Cummings JL Fairbanks LA Masterman D Miller BL Craig AH Paulsen JS Litvan I Apathy is not depression J Neuropsychiatry Clin Neurosci 1998 10 314 319 9706539 10.1176/jnp.10.3.314

[B37] Launer LJ Wind AW Deeg DJ Nonresponse pattern and bias in a community-based cross-sectional study of cognitive functioning among the elderly Am J Epidemiol 1994 139 803 812 8178793 10.1093/oxfordjournals.aje.a117077

[B38] Karpa MJ Gopinath B Beath K Rochtchina E Cumming RG Wang JJ Mitchell P Associations between hearing impairment and mortality risk in older persons: the Blue Mountains Hearing Study Ann Epidemiol 2010 20 452 459 10.1016/j.annepidem.2010.03.011 20470972

